# Biodegradation of Imazethapyr by Bacterial Strain IM9601 Isolated from Agricultural Soil

**DOI:** 10.1007/s00284-023-03533-4

**Published:** 2023-12-08

**Authors:** Zehua Xu, Baiyun Li, Yonghua Jia, Xinnian Guo, Fanyang Lv

**Affiliations:** 1 Horticultural Research Institute, Ningxia Academy of Agriculture and Forestry Sciences, Yinchuan, China; 2 Agricultural Resources and Environment Institute, Ningxia Academy of Agriculture and Forestry Sciences, Yinchuan, China; 3 Biotechnology Research Institute/State Key Laboratory of Agricultural Microbiology, Chinese Academy of Agricultural Sciences, Beijing, China

## Abstract

The widespread utilization of the herbicide imazethapyr presents significant challenges to crop rotation and results in detrimental soil degradation issues. Bacterial biodegradation has emerged as a promising and eco-friendly approach for mitigating pesticide residues contamination in the environment. In this study, a novel bacterium, identified as *Brevibacterium* sp. IM9601, was isolated and characterized based on morphological, physiological, and biochemical characteristics, as well as 16S rRNA gene sequence. This strain exhibited the ability to utilize imazethapyr as its sole carbon source for growth. Response surface methodology (RSM) was applied to optimize the degradation conditions. The most favorable conditions were determined to be a temperature of 27 °C, pH of 6.0, and an initial inoculum with a final OD_600_ of 0.15. Under these optimized condition, bacterial strain IM9601 exhibited substantial imazethapyr degradation, with removal rates of 90.08 and 87.05% for initial imazethapyr concentrations of 50 and 100 mg L^−1^, respectively, achieved within a 5-day incubation period. This investigation highlights imazethapyr-degrading capabilities of the *Brevibacterium* genus bacterial strain IM9601, marking it as a potentially novel and effective solution for addressing the environmental pollution resulting from the usage of imazethapyr. The study contributes to the growing body of research on bioremediation approaches, offering a sustainable and environmentally friendly method for mitigating the adverse impacts of herbicide contamination in agricultural settings.

**Supplementary Information:**

The online version contains supplementary material available at 10.1007/s00284-023-03533-4.

## Introduction

Herbicides are extensively employed to suppress weed growth and their applications are considered indispensable for sustaining high agricultural yields [1]. Imidazolinone herbicides constitute a heterogeneous category of agrochemicals and are designed to control weeds by inhibiting the enzyme acetohydroxyacid synthase (AHAS). They have been widely used as selective pre- or post-emergence herbicides on a variety crops, including soybean, alfalfa, wheat, and barley, since the mid-1980s [2, 3]. It has been established that imidazolinone herbicides exhibit long-lasting persistence, resulting in persistent negative impacts on the crop-rotation process and causing soil degradation problems [4]. Consequently, imidazolinone herbicides pose a continuous threat on all living species exposed to it, thereby posing significant ecological and public health concern regarding imazethapyr environment contamination [3, 5–7]. In addition to their environmental repercussions, herbicides frequently exert adverse effects on non-target wildlife species, thereby influencing the plant community diversity [8].

Imazethapyr [5-ethyl-2-(4-isopropyl-4-methyl-5-oxo-4, 5-dihydro imidazole-1H-3-yl) nicotinic acid] is a member of the imidazolinone herbicides and is primarily employed for grass and weed control in soybean cultivation, as well as other legumes crops, including barnyard grass, crabgrass, cocklebur, pigweeds, velvetleaf, and others due to its high activity at low application rates and broad spectrum of weed control [1, 9]. Previous studies have shown that imazethapyr exhibits low sorption coefficient and high solubility in water, with a various half-life ranging from 7 to 513 days depending on various factors, such as temperature, soil texture, pH, and microbial activity [10, 11].

These chemical agents block the biosynthesis of amino acids, nucleotides, and lipids or interrupt the process of photosynthesis [12, 13]. Imazethapyr undergoes slow hydrolysis and also displays toxicity toward plants and microorganisms, raising concerns about potential leaching into groundwater and its impact on ecosystem and human health [14–16]. Given its toxicity, persistent characteristics, and potential for phytotoxic effects on subsequent crops, it becomes imperative to address its removal.

Several methods, such as physical adsorption, photodegradation, and biodegradation, have been used to treat imazethapyr herbicides in recent years [17–19]. These methods play an essential role in the remediation of imazethapyr-contaminated environment. However, concerns have arisen among researchers regarding the toxicity associated with physical adsorption and photodegraded materials, like biochar, TiO2/Ti, which can lead to secondary pollution and induce oxidative stress, inflammation, and autophagy in certain organism [20–22]. In response to these concerns, there is a growing emphasis on the development of more efficient and environmentally friendly removal systems. Biodegradation has garnered increasing attention due to its eco-friendliness, high efficiency, low resource consumption, and lack of secondary pollution [18], this approach involves introducing degrading strains into the environment [1, 23, 24]. To date, several imazethapyr-degrading bacterial strains have been identified, including *Alcaligenes* sp. BH-1, *Arthrobacter crystallopoietes* WWX-1, *Achromobacter*, and *Pseudomonas* sp. IM-4 [23, 25, 26]. Notably, *Pseudomonas* sp. IM-4 exhibits the highest efficiency for imazethapyr removal, achieving a 73.4% reduction in the initially added 50-mg L^−1^ imazethapyr concentration after 7 days of inoculation. However, limited information is available regarding the optimal bacterial degradation conditions for imazethapyr. Additionally, the rate of microbial transformation is affected by soil physicochemical properties, and environmental factors [26]. Therefore, it is necessary to optimize the degradation conditions and characteristics to achieve potential maximum degradation ability.

In this study, we isolated bacterial strain IM9601 from agricultural filed soil and conducted an in-depth exploration of its imazethapyr degradation capabilities under diverse conditions, achieving an impressive degradation rate of up to 90%. The primary aim of this study is to optimize the degradation conditions, and evaluate the bioremediation potential of IM9601. The results suggest that IM9601 holds significant promise for the bioremediation of imazethapyr-contaminated environment.

## Materials and Methods

### Enrichment, Isolation, and Screening of Imazethapyr Degradation Strains

Imazethapyr (96%) and imazethapyr standard (99%) were purchased from the Institute for the Control of Agrochemicals and Sigma-Aldrich, USA, respectively. Methanol and acetonitrile were of high-performance liquid chromatography (HPLC) purity grade and other chemicals were of analytical grade. The solutions were filtered through a 0.45-μm filter membrane before injection. The mineral salt medium (MSM) which contains 1-g L^−1^NH_4_NO_3_, 1-g L^−1^ NaCl, 0.5-g L^−1^ KH_2_PO_4_, 1.5-g L^−1^K_2_HPO_4_, and 0.1-g L^−1^ MgSO_4_.7H_2_O are used for the isolation of bacterial strains. Lysogeny broth medium (LB) contains 10-g L^−1^ tryptone, 5-g L^−1^yeast extract, and 10-g L^−1^NaCl, pH 7.0–7.2, was used for the growth of bacterial strains.

Ten grams of soil samples were transferred into 250-mL erlenmeyer flasks with 100-mL sterilized MSM medium containing 50-mg L^−1^ imazethapyr. The enrichment culture was subsequently incubated in a shaking incubator at 28 °C, 160 rpm. Following a 7-day incubation period, 10 mL of aforementioned enrichment culture was transferred to 90 mL of fresh MSM medium, which contained 100-mg L^−1^ imazethapyr, and maintained under the same incubation conditions. This transfer procedure was serially conducted seven times in succession until the imazethapyr concentration reached 800 mg L^−1^. Subsequently, 200 µL of the final culture were serially diluted and plated on MSM agar plates supplemented with 100-mg L^−1^ imazethapyr as the sole carbon source. After incubation for 5 days at 28 °C, colonies were collected and purified by streaking on LB agar plates. To assess the growth and degradation capabilities of the isolated strains, these isolates were inoculated into sterilized liquid MSM medium containing 50-mg L^−1^ imazethapyr. The cultures were then incubated at 28 °C with continuous shaking at 160 rpm in dark for 7 days. The imazethapyr-degrading abilities of these isolates were determined by HPLC (Agilent 1100, Palo Alto, CA, USA) with a C18 Diamosil™, reversed-phase column. Detection was performed using an array detector at a wavelength of 258 nm, and quantification was based on retention time and the peak area of the pure standard samples [4].

### Characterization and Identification of Imazethapyr Degradation Strains

Genomic DNA was extracted according to the instructions of the Bacteria Genomic DNA Kit (Mei5bio, China) from the isolates. Subsequent to the evaluation of DNA quality via agarose gel electrophoresis, 16S rRNA gene was amplified using the universal bacterial 16S rRNA gene primers, specifically 27F (5’-AGAGTTTGATCCTGGCTCAG-3’) and 1492R (5’-TACGGTTACCTTGTTACGACTT-3’). A polymerase chain reaction (PCR) was performed using a 50-µL reaction mixture composed of 1 µL of DNA template (final concentration: 500 ng), 1 µL of each 10-µM primer stock, 25 µL 2 × Phanta Max Master Mix (Vazyme), and 22-µL sterile MilliQ H_2_O. The PCR protocol initiated with an initial denaturation step at 95 °C for 5 min, followed by 30 cycles of denaturation at 95 °C for 30 s, annealing at 58 °C for 1 min, and extension at 72 °C for 1 min. A final extension is then carried out at 72 °C for 7 min before cooling to 4 °C. A negative control reaction was performed, including all components expect the template. The resulting PCR products were purified from the gel using FastPure Gel Extraction Mini Kit (Vazyme) and subsequently submitted to Sangon Biotech (China) for sequencing. Sequences were then compared with available sequences in the EzTaxon database (http://www.ezbiocloud.net/). Phylogenetic analysis was performed using the neighbor-joining method within the software package MEGA 7.0.

The 16S rRNA genes sequences of the isolated strains in this study have been deposited with the NCBI GenBank, and their respective sequence lengths and accession numbers are presented in Table S1.

### Optimization of Imazethapyr-Degrading Conditions

Response surface methodology (RSM) based on the Box–Behnken design of experiments was employed to optimize critical parameters and their interaction, which significantly influenced the biodegradation capacity of bacterial strain IM9601 toward imazethapyr. The strain was inoculated in MSM containing imazethapyr (50 mg L^−1^) as the sole carbon source, and the samples were collected on the fourth day to assess residual imazethapyr levels. The key factors under investigation included temperature (ranging from 20 to 40 °C), medium pH (ranging from 5.0 to 9.0), and the total inoculum amount (0.1 ≤ OD_600_ ≤ 1.0). The experiment design is outlined in Table 1. The second-order polynomial equation is expressed as follows:

$${\text{Yi}} = b0 + \sum {biXi} + \sum {bijXiXj + } \sum {biiXi^{2} }$$

where ${\text{Yi}}$ is the predicted response; $Xi$ and $Xj$ are the variables (temperature, medium pH, and total inoculum amount, respectively); $b0$ is the constant; $bi$ is the linear coefficient; $bij$ is the interaction coefficient; and $bii$ is the quadratic coefficient.

**Table1:** Box–Behnken experiment design with three independent variables

Run	X_1_	X_2_	X_3_	Responses imazethapyr degradation (%)
1	0	0	0	80.61 ± 0.11
2	1	− 1	0	59.22 ± 0.33^****^
3	− 1	1	0	43.20 ± 3.19^****^
4	1	1	0	49.94 ± 1.62^****^
5	− 1	0	− 1	85.13 ± 0.50^*^
6	1	0	− 1	43.75 ± 0.97^****^
7	− 1	0	1	86.23 ± 0.42^**^
8	1	0	1	46.10 ± 2.31^****^
9	0	− 1	− 1	90.08 ± 0.27^***^
10	0	1	− 1	52.60 ± 1.03^****^
11	0	− 1	1	89.23 ± 1.76^****^
12	0	1	1	48.26 ± 2.88^****^
13	0	0	0	83.48 ± 0.69
14	0	0	0	81.71 ± 0.21
15	0	0	0	84.83 ± 0.28
16	− 1	− 1	0	89.80 ± 0.22^****^
17	0	0	0	84.44 ± 0.35

X1: Temperature (℃), − 1 (20), 0 (30), and 1 (40); X 2: pH, − 1(5), 0 (7), and 1 (9); X 3: Inoculum amount, − 1 (OD_600_ = 0.1), 0 (OD_600_ = 0.5), and 1 (OD_600_ = 1.0)

Asterisks indicate statistical significance determined using one-way ANOVA with Tukey’s post hoc test: ****P ≤ 0.0001, ***P ≤ 0.001, **P ≤ 0.01, and *P ≤ 0.05

### Biodegradation of Imazethapyr by IM9601

Bacterial strain IM9601, exhibiting the highest imazethapyr degradation ability, was selected for further studies. Degradation experiments were carried out under the optimized conditions using MSM containing 50-mg L^−1^imazethapyr as the sole carbon source, with three independent replicates. Briefly, bacterial strain IM9601 was pre-incubated in LB medium, followed by centrifugation at 8000 × g for 5 min and subsequent washing with sterile phosphate-buffered saline (PBS). The collected bacterial cells were then inoculated into 100 mL of sterile MSM medium containing 50-mg L^−1^ imazethapyr as the sole carbon source in 250-mL flasks and incubated on a shaker at 160 rpm. The residual concentration of imazethapyr was detected using HPLC as previously described. A mobile phase comprising acetonitrile/water/acetic acid (40:60:0.1, v/v/v) was used at a flow rate of 1.0 mL min^−1^. Additionally, the growth of bacterial strain IM9601 (OD_600_) was measured every 24 h. Imazethapyr biodegradation experiments involving bacterial strain IM9601 were further conducted across varying initial imazethapyr concentrations (ranging from 50 to 400 mg L^−1^) under the established optimum conditions to determine the optimum imazethapyr initial concentration.

### Statistical Analysis

Unless stated otherwise, experiments were performed three times with similar results. Each bar on graphs represents a mean of biological replicates and error bars indicate the SEM (standard error of the mean), as mentioned in figure legends for each experiment. Statistical analysis was performed using GraphPad Prism 9.0. A two-tailed unpaired Student’s *t* test with a 95% confidence interval was used. For more than two groups, one-way ANOVA with Tukey’s post hoc test was used to evaluate the difference between two groups. A probability value of *P *≤ 0.05 was considered significant. Data are presented as averages ± SEM. ****: *P *≤ 0.0001; ***:* P *≤ 0.001; **: *P *≤ 0.01; *: *P *≤ 0.05; and ns: non-significant.

## Results

### Isolation and Identification of Imazethapyr-Degrading Bacteria

Ten imazethapyr-degrading strains were isolated from MSM agar plates supplemented with imazethapyr as the sole carbon source (Table S1, Fig. S1). Imazethapyr degradation abilities of these isolates were assessed, with one strain, denoted as IM9601, exhibiting remarkable proficiency in imazethapyr degradation and consequently selected for further investigation (Table S1). The colonial morphology of bacterial strain IM9601 on the LB agar plate was circular, convex, smooth, and opaque appearance with an entire margin.

Bacterial strain IM9601 displayed positive characteristics for Gram staining, oxidase, catalase, nitrate, gelatin, and aerobic growth. However, it yielded negative results for the indole reaction, phospholipase reaction, methyl red reaction, and phenylalanine deaminase test. Furthermore, it exhibited growth potential in MSM supplemented with glucose, sucrose, or fructose, but not with D-sorbitol, arabinose, glycerol, galactose, lactose, maltose mannitol, and xylose (Table 2).

**Table 2:** Physio-biochemical and Carbon utilization test results of strain IM9601

Physio-biochemical tests	Results	Carbon utilization	Results
Gram staining	+	Glucose	+
Starch hydrolysis	+	Sucrose	+
Gelatin liquefaction	+	Fructose	+
Voges–Proskauer	+	D-Sorbitol	–
Indole reaction	–	Arabinose	–
Catalase reaction	+	Glycerol	–
Oxidase reaction	+	Galactose	–
Phospholipase reaction	–	Lactose	–
Nitrate reduction	+	Maltose	–
Methyl red reaction	–	Mannitol	–
Phenylalanine deaminase	–	Xylose	–
Production of dihydroxyacetone	–	Lactate	+

**Fig. 1:**
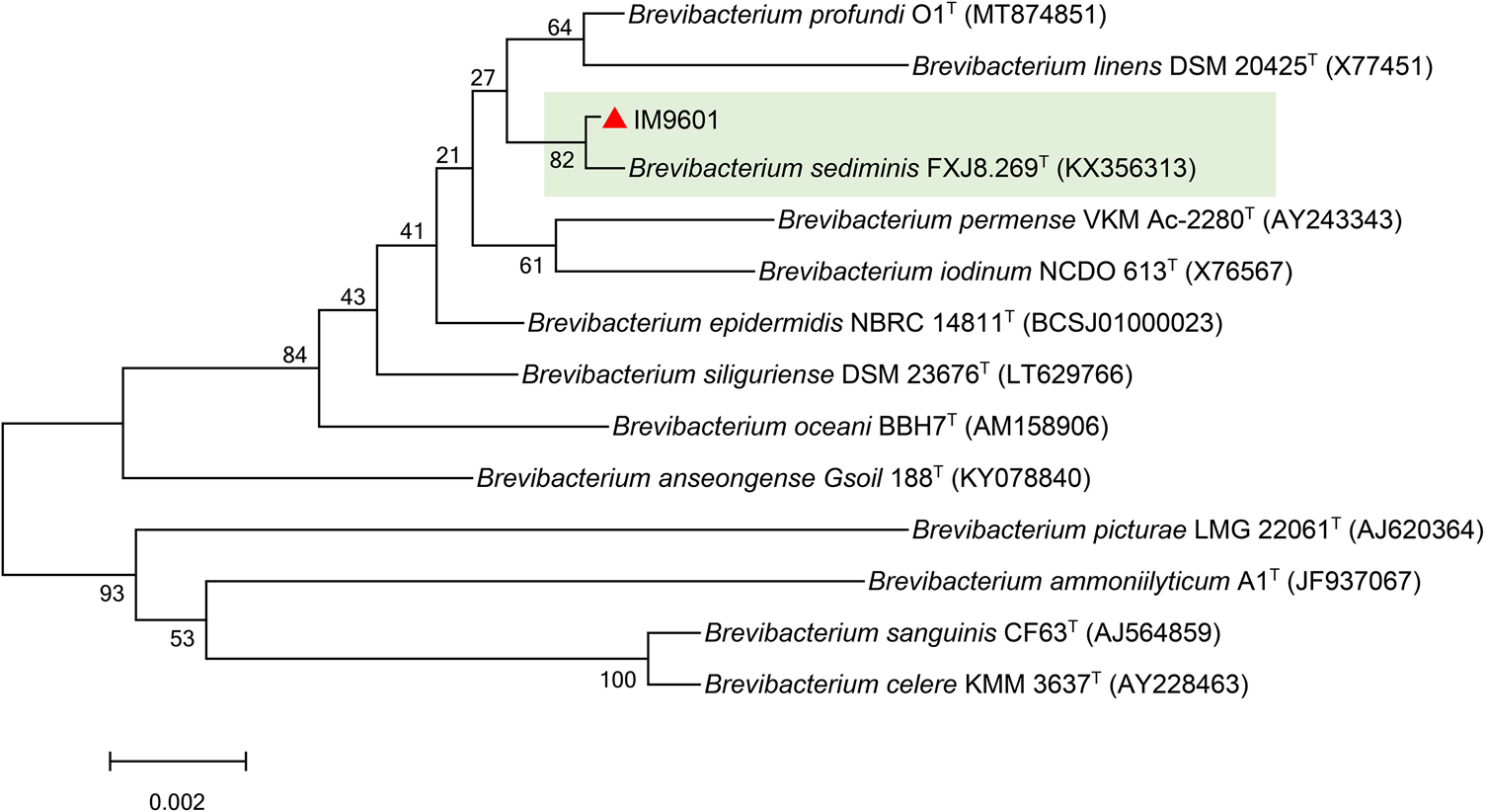
Phylogenetic tree based on the 16S rRNA gene sequences of IM9601 (KX098473) and type strains. Numbers in parentheses represent the GenBank accession numbers of the sequences

The 16S rRNA gene sequence of bacterial strain IM9601 was meticulously determined and subsequently deposited in the GenBank database (accession number: KX098473). Phylogenetic analysis revealed that IM9601 was a member of the *Brevibacterium* genus, showing the closest genetic relationship with *Brevibacterium sediminis* FXJ8.267^ T^ (KX356313) and *Brevibacterium iodinum* NCDO 613^ T^ (X76567) (Fig. 1). The sequence homology between 16S rRNA genes of IM9601 and those of *Brevibacterium sediminis* (KX356313) and *Brevibacterium iodinum* (KX356313) was 99.39 and 99.18%, respectively. This study underscores the capacity of a *Brevibacterium* sp. strain to effectively degrade imazethapyr, offering a promising solution for addressing imazethapyr-caused environmental pollution.

### Optimizing Imazethapyr-Degrading Conditions of *Brevibacterium* sp. Strain IM9601

**Fig. 2:**
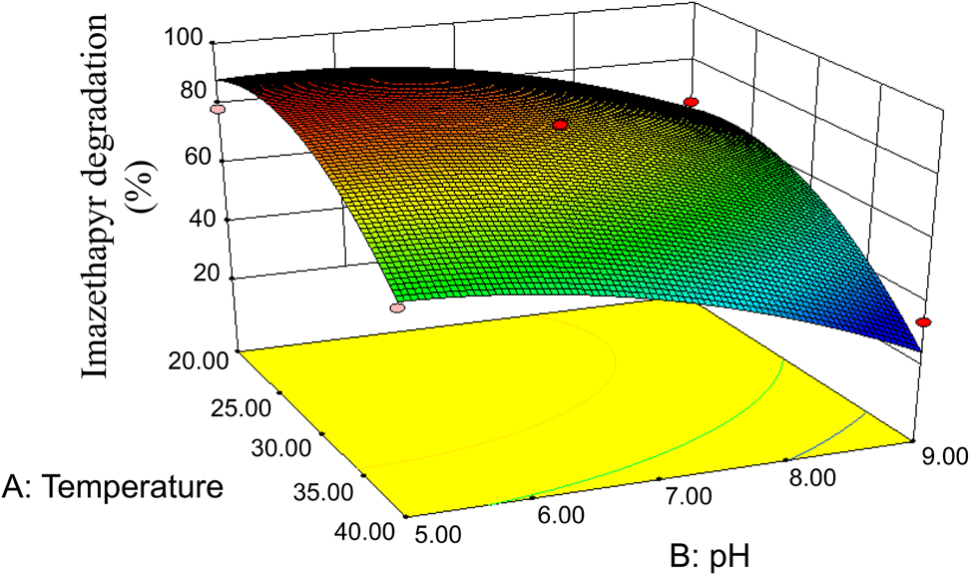
Response surface curves showing the effects of medium temperature and pH on imazethapyr biodegradation with the total biomass amount of bacterial strain IM9601

The Box–Behnken design methodology was used to analyze the primary and interactive effects of pivotal variables that significantly affect the imazethapyr degradation capacity exhibited by *Brevibacterium* sp. strain IM9601. These crucial variables encompassed incubation temperature (ranging from 20 to 40 °C), medium pH (with a range of 5–9), and the quantity of inoculum (0.1 ≤ OD_600_ ≤ 1.0). The designed matrix and response values are listed in Table 1. Statistical analysis to experimental data was performed with the SAS software package, integrated within the Design-Expert (8.0.6) program. The analysis yielded a second-degree polynomial equation, which was formulated to encapsulate the imazethapyr degradation rate of *Brevibacterium* sp. strain IM9601 as follows:

$$\begin{gathered} {\text{Y}} = {83}{\text{.01 - 13}}{\text{.17X1 - 17}}{\text{.42X2 - 0}}{\text{.34X3}} + {9}{\text{.33X1X2}} + {0}{\text{.31X1X3}} \hfill \\ { - 0}{\text{.62X2X3 - 14}}{\text{.23X1}}^{{2}} { - 8}{\text{.24X2}}^{{2}} - 3.48X3^{2} \hfill \\ \end{gathered}$$

where Y represents the imazethapyr degradation rate of bacterial strain IM9601, while X_1_, X_2_, and X_3_ symbolize the coded variables for the incubation temperature, medium pH, and inoculums quantity, respectively. The regression analysis results showed that temperature (X_1_) and medium pH (X_2_) were deemed as essential factors (*P *≤ 0.05), whereas the linear and quadratic terms of inoculums quantity (X_3_), along with the interaction terms, were deemed statistically insignificant (*P *> 0.05). In particular, under the specified inoculant amount of OD_600_ = 0.15, the response surface was constructed to visually represent the combined effects of temperature and medium pH on the imazethapyr degradation rate of *Brevibacterium* sp. strain IM9601. Figure 2 shows the optimal conditions for imazethapyr degradation by IM9601, which were determined to be a temperature of 27 °C and pH of 6 (Fig. 2). By maintaining a constant inoculum quantity, a three-dimensional response surface was constructed to elucidate the intricate relationship between incubation temperature and pH in the context of imazethapyr biodegradation exhibited by *Brevibacterium* sp. strain IM9601.

### Bacterial Growth and Degradation Ability of *Brevibacterium* sp. Strain IM9601

**Fig. 3:**
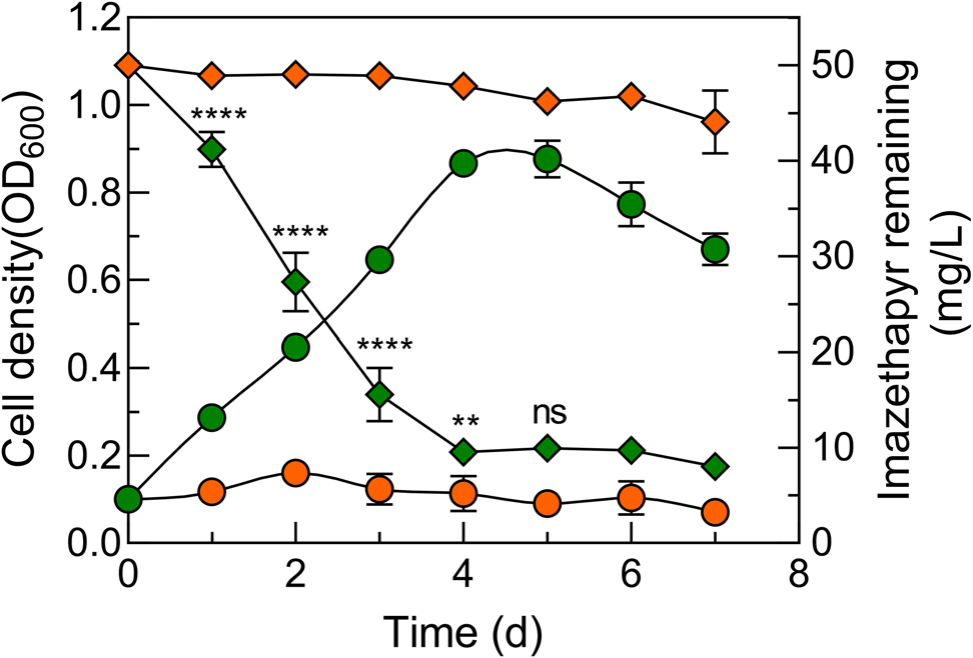
Growth and degradation of bacterial strain IM9601 in imazethapyr during biodegradation studies. Symbol legend: ♦, degradation kinetics in MSM supplemented with imazethapyr as the sole carbon source (green) and non-inoculated control (orange); ●, growth curve in MSM supplemented with imazethapyr as the sole carbon source (green) and non-inoculated control (orange). Significance determined using one-way ANOVA with Tukey’s post hoc test: ****P ≤ 0.0001, **P ≤ 0.01, and ns: non-significant. Data are expressed as the mean of three replicates with standard deviation (Color figure online)

Degradation and growth kinetics of *Brevibacterium* sp. strain IM9601 were tested within a milieu of varying imazethapyr concentrations in MSM, wherein imazethapyr served as the sole carbon source. Under the optimal conditions (pH of 6, incubation temperature of 27 °C, and cell density of OD_600_ = 0.15), imazethapyr degradation exceeding 90% was achieved within a span of 5 days, commencing with an initial imazethapyr concentration of 50 mg L^−1^ (Fig. 3). Notably, the peak of imazethapyr degradation was reached on the fourth day. In stark contrast, non-inoculated cultures displayed no discernible alterations in imazethapyr concentration. Moreover, the process of imazethapyr degradation was concomitantly correlated with a notable augmentation in cell density, as evidenced by an increase in OD_600_ from approximately 0.1–1.0 (Table 1). These data indicated that *Brevibacterium* sp. strain IM9601 efficiently utilizes imazethapyr as its carbon source, thus enabling substantial imazethapyr degradation.

**Fig. 4:**
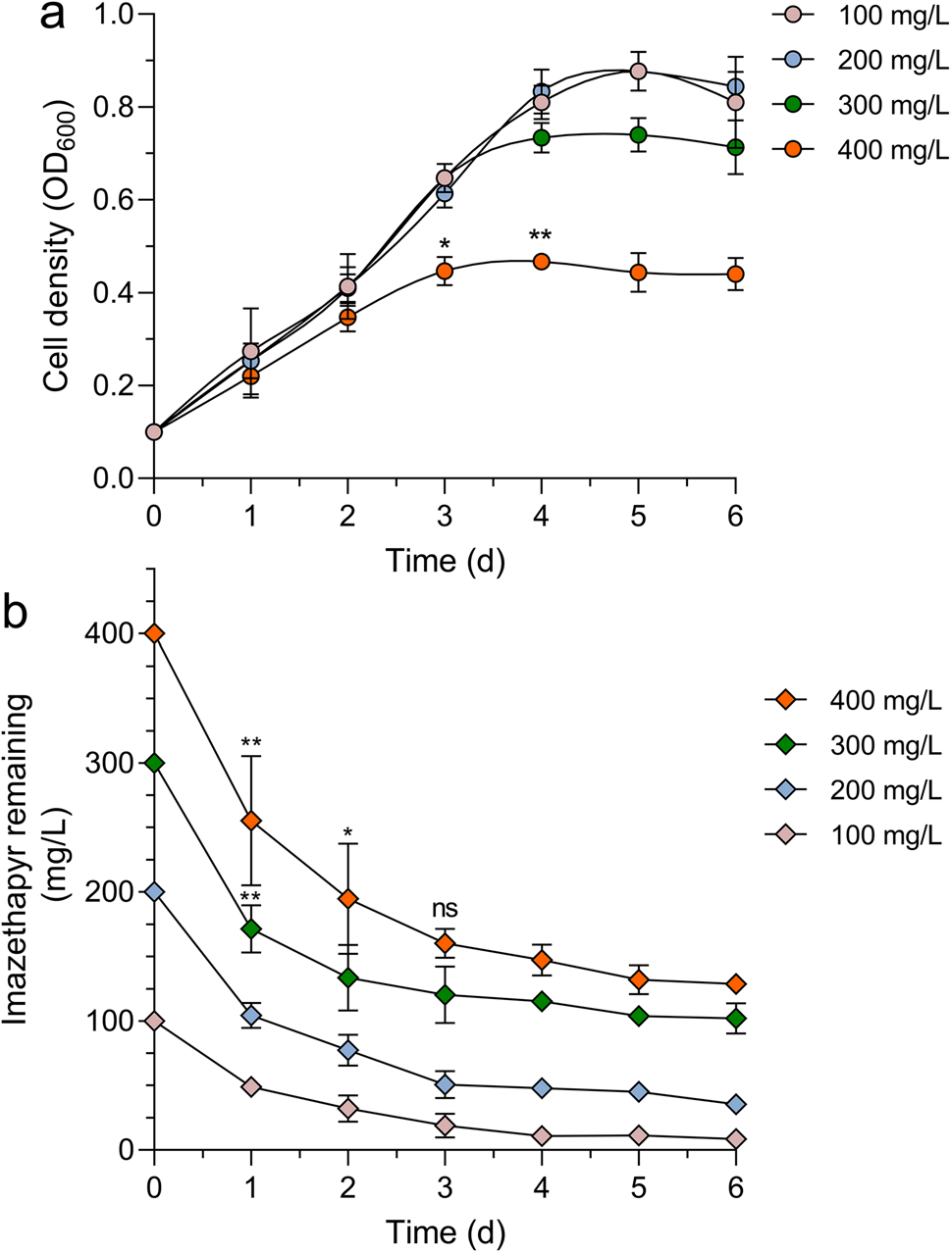
Growth curves (**a**) and degradation kinetics (**b**) of bacterial strain IM9601 on the different initial imazethapyr concentrations. Asterisks indicate statistical significance determined using one-way ANOVA with Tukey’s post hoc test: **P ≤ 0.01, *P ≤ 0.05, and ns: non-significant. Data are expressed as the mean of three replicates with standard deviation

To further evaluate the effects of initial imazethapyr concentration on imazethapyr degradation efficiency exhibited by *Brevibacterium* sp. strain IM9601, growth curves and degradation kinetics of *Brevibacterium* sp. strain IM9601 were assessed across a range of initial imazethapyr concentration spanning from 100 to 400 mg L^−1^ (Fig. 4a and b). Results indicated that *Brevibacterium* sp. strain IM9601 could attain imazethapyr degradation levels of up to 87.05% when initial concentrations were set at 100 mg L^−1^ over a 5-day incubation period. However, a noticeable decline in degradation rate was observed at initial concentrations of 200 and 300 mg L^−1^ (Fig. 4b), with a substantial reduction to 33.56% recorded at an initial concentration of 400 mg L^−1^. Further studies on imazethapyr degradation in aqueous and soil environments are necessary for practical applications.

## Discussion

Imazethapyr is wildly used for cultivating rice, soybeans, and other crop plants [27]. However, this extensive usage has been linked to adverse consequences, including the disruption of natural soil biodiversity and the inhibition of growth of successive crop rotations [28]. Consequently, there exists a pressing need to develop sustainable and efficacious methods for the removal of these environmentally detrimental substances. One such solution involves the utilization of specific bacteria capable of employing herbicides as their sole carbon and nitrogen sources for growth [29]. In this study, a remarkably successful strain IM9601 was isolated from agricultural soils and was confirmed to be able to utilize imazethapyr as a sole carbon source for growth. Analyses of the16S rRNA gene sequence, as well as the morphological and physio-biochemical characteristics, have conclusively placed IM9601 within the *Brevibacterium* genus. Notably, this is the first report which showed that *Brevibacterium* can effectively utilize imazethapyr as a sole carbon source for growth and could achieve imazethapyr degradation levels of up to 90%. This level of degradation is significantly superior when compared to previously reported bacteria, such as *Arthrobacter crystallopoietes* (which can utilize imazethapyr as a sole carbon source for growth) and *Pseudomonas* sp. (capable of degrading up to 62% of imazethapyr) and were employed for imazethapyr degradation [23, 25].

Microbial growth is contingent upon the availability of essential nutrients, primarily carbon, and nitrogen sources, within the surrounding environment. Bacteria have demonstrated the capability to utilize herbicides as their exclusive carbon and nitrogen sources for growth, and prior research has investigated the influence of carbon and nitrogen sources on herbicide biodegradation [30, 31]. In this study, the growth and degradation ability of strain IM9601 were tested in MSM medium containing varying concentrations of imazethapyr. As illustrated in Fig. 4, it is showed that elevated concentration of imazethapyr and low degradation results were obtained by IM9601 which is consistent with previous findings indicating that an increase in imazethapyr concentration lead to a reduction in degradation efficiency [22]. This decline in degradation efficiency at higher imazethapyr concentration may be attributed to the toxic effects exerted by elevated imazethapyr concentration on the bacterial population. It is worth noting that the concentrations of imazethapyr encountered in crop production areas are considerably lower than the highest concentration examined in this study [7].

Biodegradation is widely recognized as an environmentally sustainably approach for the eliminate of herbicides, and microbial remediation represents a potent tool in this endeavor [31–33]. Nevertheless, the intricate interplay of infinite interactions of environmental factors, including pH and temperature, significantly impacts the activity of soil microorganisms, which have been recommended as determining factor for the biodegradation practicability [34–37]. The principal objective of the present study was to optimize the degradation conditions that may affect the biodegradation capacity of strain IM9601. Employing response surface methodology based on the Box–Behnken design, we systematically explored the interactive and concurrent effector of pH, temperature, and cell concentration on the percentage of imazethapyr degradation. The results show that maximum imazethapyr degradation was achieved under condition featuring a medium pH of 6.0, a temperature of 27 °C, and inoculum amount (OD_600_) of 0.15. The second-degree polynomial equation was derived and subsequently validated using experimental values at the identified optimal conditions. In the context of bacterial-mediated herbicide degradation, pH tolerance emerges as a crucial factor. In consonance with our findings, numerous herbicide-degrading bacteria have been reported to operate efficiently within a pH range spanning 6.0 to 8.0, as observed in the case of Sphingomonadaceae and Vicinamibacteraceae [38, 39]. Temperature represents another key factor governing herbicides biodegradation [39]. To examine the effect of temperature on imazethapyr degradation of strain IM9601, a series of experiments were performed across a wide temperatures range of 20 °C to 40 °C. The result indicated that IM9601 exhibited a higher degradation ability at lower temperatures. The observed inhibition of imazethapyr degradation at elevated temperatures may be attributed to the reduce viability of bacteria cells. The results in our study show similar investigations conducted by other researchers employing RSM for optimization [40, 41].

## Conclusion

This study has highlighted the remarkable imazethapyr-degrading capabilities of the *Brevibacterium* sp. strain IM9601, offering a potential innovative and effective solution for addressing environmental pollution resulting from the use of imazethapyr. The research significantly contributes to the expanding body of microbial resources concerning bioremediation approaches, providing a sustainable and ecologically sound approach to mitigating the detrimental effect of herbicide pollution in agricultural ecosystems. However, it is essential to emphasize the need for further investigations into imazethapyr degradation in diverse environments and practical applications to fully exploit the potential of *Brevibacterium* sp. strain IM9601 for bioremediation purposes. Hence, it holds significant importance to conduct additional research aimed at elucidating the application of the imazethapyr-degrading bacterium in environments or farmlands contaminated with imazethapyr. This endeavor is vital for a comprehensive assessment of their application in fostering a more eco-friendly and sustainable approach to the commercial production of imazethapyr-degrading bacteria.

### Supplementary Information

Below is the link to the electronic supplementary material.

Supplementary file1 (DOCX 725 KB)

## Data Availability

All data generated or analyzed during this study are included in this published article.

## Code Availability

No applicable.

## References

[1] Tan S, Evans RR, Dahmer ML (2005). Imidazolinone-tolerant crops: history, current status and future. Pest Manag Sci.

[2] Espy R, Pelton E, Opseth A (2011). Photodegradation of the herbicide imazethapyr in aqueous solution: effects of wavelength, pH, and natural organic matter (NOM) and analysis of photoproducts. J Agric Food Chem.

[3] Pérez-Iglesias JM, Soloneski S, Nikoloff N (2015). Toxic and genotoxic effects of the imazethapyr-based herbicide formulation Pivot H® on montevideo tree frog *Hypsiboas pulchellu*s tadpoles (Anura, Hylidae). Ecotoxicol Environ Saf.

[4] Goetz AJ, Lavy TL, Gbur EE (1990). Degradation and field persistence of imazethapyr. Weed Sci.

[5] Pérez-Iglesias JM, Fanali LZ, Franco-Belussi L (2021). Multiple level effects of imazethapyr on *Leptodactylus latinasus* (Anura) adult frogs. Arch Environ Contam Toxicol.

[6] Pérez-Iglesias JM, Franco-Belussi L, Moreno L (2016). Effects of glyphosate on hepatic tissue evaluating melanomacrophages and erythrocytes responses in neotropical anuran *Leptodactylus latinasus*. Environ Sci Pollut Res Int.

[7] Pérez-Iglesias JM, Ruiz de Arcaute C, Natale GS (2017). Evaluation of imazethapyr-induced DNA oxidative damage by alkaline Endo III- and Fpg-modified single-cell gel electrophoresis assay in *Hypsiboas pulchellus* tadpoles (Anura, Hylidae). Ecotoxicol Environ Saf.

[8] Qi Y, Li J, Guan X (2020). Effects of herbicides on non-target plant species diversity and the community composition of fallow fields in northern China. Sci Rep.

[9] Liu L, Feng F, Chin Paau M (2015). Sensitive determination of kaempferol using carbon dots as a fluorescence probe. Talanta.

[10] Wu H, He X, Dong H (2017). Impact of microorganisms, humidity, and temperature on the enantioselective degradation of imazethapyr in two soils. Chirality.

[11] Magdaleno A, Peralta Gavensky M, Fassiano AV (2015). Phytotoxicity and genotoxicity assessment of imazethapyr herbicide using a battery of bioassays. Environ Sci Pollut Res Int.

[12] Tajnaiová L, Vurm R, Kholomyeva M (2020). Determination of the ecotoxicity of herbicides roundup® classic pro and garlon new in aquatic and terrestrial environments. Plants.

[13] Saha A, Bhaduri D, Pipariya A, Jain NK (2016). Influence of imazethapyr and quizalofop-p-ethyl application on microbial biomass and enzymatic activity in peanut grown soil. Environ Sci Pollut Res Int.

[14] Kaur P, Bhatia A, Kaur H, Bhullar MS (2023). Adsorption and desorption of Imazethapyr in Indian soils in relation to soil properties and temperature. Int J Environ Sci Technol.

[15] McGinley J, Healy MG, Ryan PC (2023). Impact of historical legacy pesticides on achieving legislative goals in Europe. Sci Total Environ.

[16] Pileggi M, Pileggi SAV, Sadowsky MJ (2020). Herbicide bioremediation: from strains to bacterial communities. Heliyon.

[17] Yavari S, Sapari NB, Malakahmad A (2020). Adsorption–desorption behavior of polar imidazolinone herbicides in tropical paddy fields soils. Bull Environ Contam Toxicol.

[18] Tan H, Kong D, Li Q (2022). Metabolomics reveals the mechanism of tetracycline biodegradation by a *Sphingobacterium mizutaii* S121. Environ Pollut.

[19] Wang J, Yuan M, Cao N (2023). In situ boron-doped cellulose-based biochar for effective removal of neonicotinoids: adsorption mechanism and safety evaluation. Int J Biol Macromol.

[20] Isak N, Xhaxhiu K (2023). A review on the adsorption of diuron, carbaryl, and alachlor using natural and activated clays. Remediation.

[21] Singh SK, Mishra PK, Upadhyay SN (2023). Recent developments in photocatalytic degradation of insecticides and pesticides. Rev Chem Eng.

[22] Huang X, Pan J, Liang B (2009). Isolation, characterization of a strain capable of degrading imazethapyr and its use in degradation of the herbicide in soil. Curr Microbiol.

[23] Jin HM, Choi EJ, Jeon CO (2013). Isolation of a BTEX-degrading bacterium, *janibacter* sp. sb2, from a sea-tidal flat and optimization of biodegradation conditions. Bioresour Technol.

[24] Wang X, Liu X, Wang H, Dong Q (2007). Utilization and degradation of imazaquin by a naturally occurring isolate of *Arthrobacter crystallopoietes*. Chemosphere.

[25] Chen D, Liu SJ, Du W (2019). Chemotactic screening of imidazolinone-degrading bacteria by microfluidic SlipChip. J Hazard Mater.

[26] Pertile M, Antunes JEL, Araujo FF (2020). Responses of soil microbial biomass and enzyme activity to herbicides imazethapyr and flumioxazin. Sci Rep.

[27] Gehrke VR, Fipke MV, de Avila LA, Camargo ER (2021). Understanding the opportunities to mitigate carryover of imidazolinone herbicides in lowland rice. Agriculture.

[28] Shivani GSK, Gill RK (2023). Effect of herbicide stress on synchronization of carbon and nitrogen metabolism in lentil (*Lens culinaris Medik*.). Plant Physiol Biochem.

[29] Vercellino M, Gómez MA (2013). Denitrifying capacity of rhizobial strains of argentine soils and herbicide sensitivity. Ann Microbiol.

[30] Phugare SS, Jadhav JP (2015). Biodegradation of acetamiprid by isolated bacterial strain Rhodococcus sp. BCH2 and toxicological analysis of its metabolites in silkworm (Bombax mori). Clean: Soil, Air, Water.

[31] Lei Q, Zhong J, Chen SF (2023). Microbial degradation as a powerful weapon in the removal of sulfonylurea herbicides. Environ Res.

[32] Semple KT, Reid BJ, Fermor TR (2001). Impact of composting strategies on the treatment of soils contaminated with organic pollutants. Environ Pollut.

[33] Dror I, Yaron B, Berkowitz B (2017). Microchemical contaminants as forming agents of anthropogenic soils. Ambio.

[34] Chianese S, Fenti A, Iovino P (2020). Sorption of organic pollutants by humic acids: a review. Molecules.

[35] Khatoon H, Rai JPN (2020). Optimization studies on biodegradation of atrazine by Bacillus badius ABP6 strain using response surface methodology. Biotechnol Rep.

[36] Ahmad F, Anwar S, Firdous S (2018). Biodegradation of bispyribac sodium by a novel bacterial consortium BDAM: optimization of degradation conditions using response surface methodology. J Hazard Mater.

[37] Cheng L, Wang L, Wang X (2023). The various effect of cow manure compost on the degradation of imazethapyr in different soil types. Chemosphere.

[38] Wen Y, Lichtfouse E, Sharma VK, Ma X (2023). Overlooked involvement of phosphate radicals in the degradation of the atrazine herbicide by sulfate radical-based advanced oxidation. Environ Chem Lett.

[39] Muskus AM, Krauss M, Miltner A (2020). Degradation of glyphosate in a colombian soil is influenced by temperature, total organic carbon content and pH. Environ Pollut.

[40] Popa Ungureanu C, Favier L, Bahrim G, Amrane A (2014). Response surface optimization of experimental conditions for carbamazepine biodegradation by Streptomyces MIUG 4.89. New Biotechnol.

[41] Guo Y, Dong S, Zhou D (2022). Optimization of the photocatalyst coating and operating conditions in an intimately coupled photocatalysis and biodegradation reactor: towards stable and efficient performance. Environ Res.

