# Supplementary material for: Biodegradation of Imazethapyr by Bacterial Strain IM9601 Isolated from Agricultural Soil
Source: Curr Microbiol. 2023 Dec 8;81(1):33. doi: 10.1007/s00284-023-03533-4 (PMC10703984; doi:10.1007/s00284-023-03533-4)
Supplement: Supplementary file 1 — Supplementary file1 (DOCX 725 KB) [file 284_2023_3533_MOESM1_ESM.docx]

**Supplemental Data**

**Biodegradation of Imazethapyr by Bacterial Strain IM9601 Isolated from Farmland**

Current Microbiology

Zehua Xu^1^, Baiyun Li^1^, Yonghua Jia^1^, Xinnian Guo^2^ & Fanyang Lv^3^*

^1^Horticultural Research Institute, Ningxia Academy of Agriculture and Forestry Sciences, Yinchuan, China.

^2^Agricultural Resources and Environment Institute, Ningxia Academy of Agriculture and Forestry Sciences, Yinchuan, China.

^3^Biotechnology Research Institute/State Key Laboratory of Agricultural Microbiology, Chinese Academy of Agricultural Sciences, Beijing, China

*Correspondence: lvfanyang@caas.cn (F. L.)


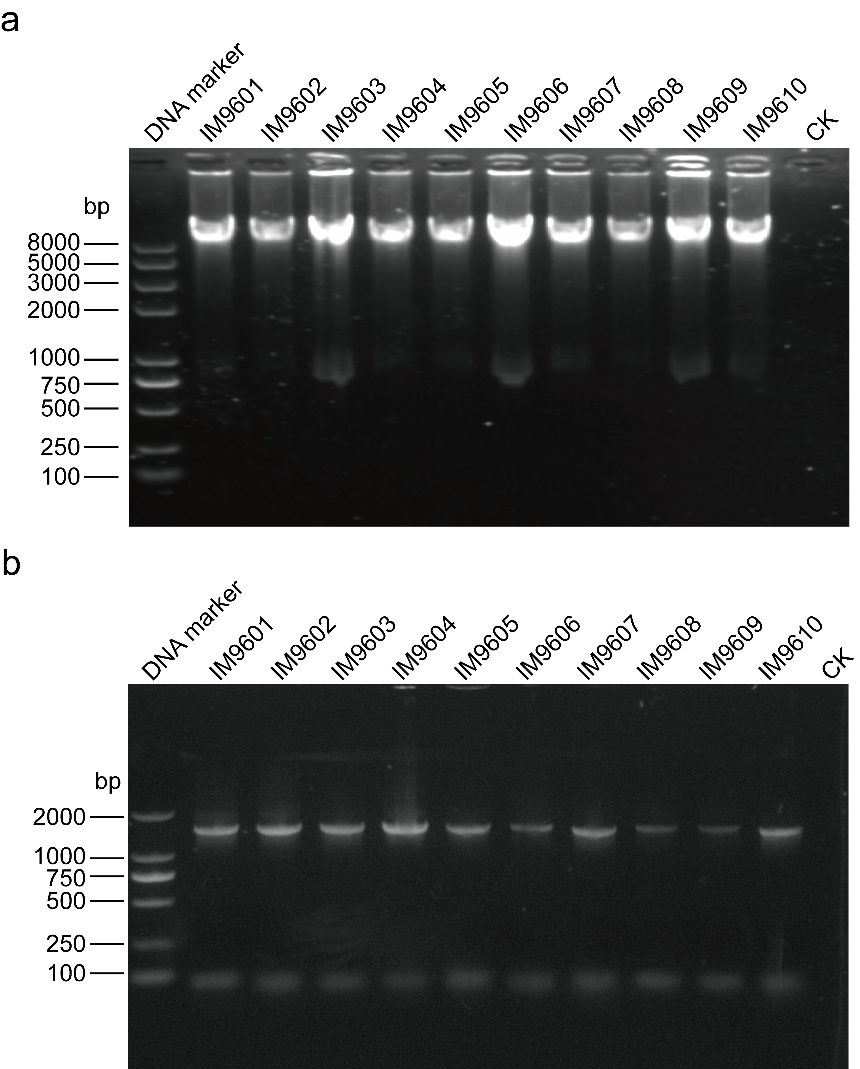


Figure S1. Gel electrophoresis (0.8 % agarose) of DNA extracted from imazethapyr-degrading bacteria (a) and gel electrophoresis (1 % agarose) of their 16S rRNA gene PCR products (b).

| Strains | Accession  Number | Length  (bp) | Sequence Homology | | | Imazethapyr-degrading rate (%) | |
| --- | --- | --- | --- | --- | --- | --- | --- |
|  |  |  | Description | Identity (%) | |  |  |
| IM9601 | KX098473.1 | 1359 | *Brevibacterium epidermidis* NCDO 2286^T^ (X76565) | | 99.19 | | 74.9 ± 8.1 |
| IM9602 | OR534292 | 1348 | *Pseudoxanthomonas spadix* IMMIB AFH-5^T^ (AM418384) | | 97.85 | | 60.5 ± 1.3 |
| IM9604 | OR534293 | 1370 | *Microbacterium arborescens* DSM 20754^T^ (X77443) | | 99.93 | | 49.3 ± 27.6^*^ |
| IM9605 | OR534300 | 1393 | *Microbacterium testaceum DSM 20166*^T^ (X77445) | | 98.71 | | 39.7 ± 13.3^*^ |
| IM9606 | OR534294 | 1280 | *Rhizobium massiliae* 90A^T^ (AF531767) | | 99.9 | | 36.2 ± 5.4^***^ |
| IM9607 | OR534295 | 1357 | *Rhodococcus jialingiae* djl-6-2 ^T^ (DQ185597) | | 99.78 | | 35.3 ± 13.1^**^ |
| IM9608 | OR534296 | 1389 | *Pseudomonas nitritireducens* WZBFD3-5A2^T^ (HM246143) | | 99.86 | | 30.2 ± 9.6^**^ |
| IM9609 | OR534297 | 1343 | *Rhodococcus baikonurensis* GTC 1041^T^ (AB071951) | | 99.19 | | 23.5 ± 2.6^***^ |
| IM9610 | OR534298 | 1330 | *Bosea thiooxidans* DSM 9653^T^ (AJ250796) | | 99.92 | | 17.6 ± 3.1^***^ |
| IM9611 | OR534299 | 1389 | *Pseudomonas nitroreducens* DSM 14399^T^ (AM088474) | | 100 | | 6.2 ± 0.2^****^ |

Table S1. NCBI GenBank accession numbers for the nucleotide sequences of the 16S rRNA gene of imazethapyr-degrading strains isolated in this study and their degradation rate.

Asterisks indicate statistical significance determined by one-way ANOVA with Tukey’s post hoc test: ****: *p* ≤ 0.0001; ***: *p* ≤ 0.001, ** *p*≤ 0.01, * *p*≤ 0.05.
